# Changes in antioxidant system and sucrose metabolism in maize varieties exposed to Cd

**DOI:** 10.1007/s11356-022-20422-8

**Published:** 2022-04-28

**Authors:** Cong Li, Yingdi Cao, Tianfeng Li, Meiyu Guo, Xinglin Ma, Yanshu Zhu, Jinjuan Fan

**Affiliations:** 1 College of Bioscience and Biotechnology, Shenyang Agricultural University, Shenyang Key Laboratory of Maize Genomic Selection Breeding, Shenyang, 110866 China; 2 Institute of Crop Science, Chinese Academy of Agricultural Sciences (CAAS), Beijing, 100081 China

**Keywords:** Cadmium stress, Antioxidant systems, Sucrose metabolism, Maize, Phytohormone, Seed germination

## Abstract

Different maize varieties respond differentially to cadmium (Cd) stress. However, the physiological mechanisms that determine the response are not well defined. Antioxidant systems and sucrose metabolism help plants to cope with abiotic stresses, including Cd stress. The relationship of these two systems in the response to Cd stress is unclear. Seed is sensitive to Cd stress during germination. In this study, we investigated changes in the antioxidant system, sucrose metabolism, and abscisic acid and gibberellin concentrations in two maize varieties with low (FY9) or high (SY33) sensitivities to Cd under exposure to CdCl_2_ (20 mg L^−1^) at different stages of germination (3, 6, and 9 days).The seed germination and seedling growth were inhibited under Cd stress. The superoxide, malondialdehyde, and proline concentrations, and the superoxide dismutase, peroxidase, catalase, and lipoxygenase activities increased compared with those of the control (CK; without Cd). The expression levels of three genes (*ZmOPR2*, *ZmOPR5*, and *ZmPP2C6*) responsive to oxidative stress increased differentially in the two varieties under Cd stress. The activity of the antioxidant system and the transcript levels of oxidative stress–responsive genes were higher in the Cd-tolerant variety, FY9, than in the sensitive variety, SY33. Sucrose metabolism was increased under Cd stress compared with that of the CK and was more active in the Cd-sensitive variety, SY33. These results suggest that the antioxidant system is the first response to Cd stress in maize, and that sucrose metabolism is cooperative and complementary under exposure to Cd.

**Supplementary Information:**

The online version contains supplementary material available at 10.1007/s11356-022-20422-8.

## Introduction

About 15,000 t cadmium (Cd) is released into the environment each year from human industrial activities between 2001 and 2004, and increased gradually to 22,200 t in 2014 (USGS 2015). Environmental Cd is harmful to humans because of its high toxicity and high solubility in water (Guo et al. 2014). Cadmium is absorbed easily by crop roots and is transported throughout the plant, including to the seed, and, once in the food chain, poses a threat to human health (Satohnagasawa et al. 2012). Accumulation of Cd in plant tissues causes inhibition of photosynthesis, decrease in biomass production and yield, and potentially, plant death (Skorzynskapolit et al. 2010; Zhang et al. 2013).

In plants, the mechanisms to overcome Cd stress are complex and can be classified into different strategies (Shahid et al. 2016). The Cd absorbed from the environment is first deposited in the cell wall. The Cd that enters the protoplasm is chelated by organic acids and phytochelatins (Keltjens and Beusichem 1998; Radhouane et al. 2006). The Cd–phytochelatin compounds are transported to and stored in the vacuole to alleviate Cd stress. This may be a mechanism by which plants avoid Cd stress. Cadmium is transported in plants mainly by competing for transporters with other divalent metal ions, including manganese, calcium, and iron (Zeng et al. 2017). In plant cells, Cd was first sequestrated within vacuoles and chelation (Hossain et al. 2012). Meanwhile, free Cd ions induce the production of reactive oxygen species (ROS), including singlet oxygen (^1^O_2_), superoxide (O_2_^•−^), hydrogen peroxide (H_2_O_2_), and hydroxyl radicals (OH^−^), which can impair redox homeostasis, leading to peroxidation of membrane fatty acids and enzyme inactivation (Sharma and Dietz 2009; Gill and Tuteja 2010). Many metabolism systems activated in response to Cd stress are also important defense mechanisms. Through evolution and artificial selection, plants have developed multifarious strategies to cope with accumulation of ROS under Cd stress. For example, plants use the antioxidant systems to regulate their response to Cd stress (Mittler et al. 2004; Rehman et al. 2019). Antioxidant enzymes, such as superoxide dismutase (SOD), peroxidase (POD), and catalase (CAT), scavenge and decompose ROS to maintain the redox in a steady state (Moller et al. 2007). Isoforms of SOD coupled with metal elements, such as copper, zinc, iron, or manganese, and located in either the cytoplasm, chloroplasts, or mitochondria disproportionate O_2_^•−^ to produce H_2_O_2_. The enzymes POD and CAT show an affinity for H_2_O_2_, which is degraded to H_2_O and O_2_ (Elstner 1982; Bowler et al. 1992; Foyer et al. 1994). The transcript levels of antioxidative enzymes are affected by the response of plants to oxidative stress. Free L-proline is a non-enzyme antioxidant that accumulates in plants stressed by heavy metals (Sharma and Dietz 2006). Proline reacts with OH^−^ under H abstraction and quenches ^1^O_2_ that is generated from the photochemical reaction (Rustgi et al. 1977; Alia and Matysik 2001). Proline can reduce the toxicity of ROS by scavenging ^1^O_2_ and OH^−^ to protect the cellulose backbone (Kaul et al. 2008).

Seed is highly sensitive to environmental factors during germination, the first phase in the life cycle (Jisha et al. 2013). Germination begins when the dry seed absorbs water. The reserves in the seed are mobilized to drive different metabolic processes (Bewley 1997). Stresses induced by heavy metals can inhibit or delay seed germination and impair the establishment of seedlings (Ahmad et al. 2012). Previous studies reported that the redox balances and mobilization of reserves could be disturbed if the seed experiences Cd stress during germination (Kuriakose and Prasad 2008; Junyu et al. 2008). Seed germination is also regulated by plant hormones, such as abscisic acid (ABA) and gibberellic acid (GA). These two hormones play antagonistic roles in the regulation of seed germination (Kucera et al. 2005; Holdsworth et al. 2008): ABA maintains dormancy and inhibits germination, whereas GA regulates dormancy release and promotes germination. These two hormones are also involved in the signal transduction pathways of abiotic stresses, and the contents of ABA and GA may change in plants that are exposed to Cd or other heavy metals (Guo et al. 2019).

Sucrose metabolism also plays an important role in plant growth, development, and response to stress. Metabolites such as sucrose, glucose, fructose, and other soluble sugars, which are produced during sucrose metabolism, are directly or indirectly involved in protecting plants from abiotic stresses, for example, as signal molecules that regulate gene expression and osmotic protectants that protect biomolecules and membranes (Ruan 2014). The metabolite concentrations in plants may change under Cd stress (Verma and Dubey 2001; Sfaxibousbih et al. 2010).

Different accessions of the same crop may respond differentially to Cd stress. Researchers have noted that the amounts of Cd that accumulate, and the responses to Cd, vary considerably among different varieties of several crops (Liu et al. 2007; Guo et al. 2019). Maize (*Zea mays* L.) is an important grain crop that is grown worldwide. Cadmium impairs the growth of maize, leading to decreased yields (Zhao et al. 2018). The effects of Cd on different maize varieties have been studied by various researchers; for example, Zhao et al. (2018) analyzed the genetic structure of Cd accumulation in 269 maize accessions.

Sensitivity to Cd stress is greater during seed germination than during seedling establishment. Sucrose and the substrate of sucrose metabolism are mainly derived from photosynthesis in the seedling stage, whereas these compounds are derived from the decomposition products of stored substances in grains during germination. In previous research, we observed that sucrose metabolism that protects maize plants from Cd stress was more active in variety SY33 than in variety FY9 at the seedling stage (Li et al. 2020). However, the underlying physiological mechanisms that help to mitigate and alleviate Cd toxicity during germination of different varieties of maize are not completely understood. The responses to Cd toxicity in plants are complex processes involving multiple systems. Therefore, in this study we investigated the effect of Cd on two varieties of maize, namely, a Cd-tolerant variety (FY-9) and a Cd-sensitive variety (SY33), during the germination phase, and evaluated how antioxidant systems and sucrose metabolism interacted to alleviate Cd toxicity.

## Materials and methods

### Plant material, growth conditions, and Cd treatments

Seeds of the maize varieties SY33 and FY9 were obtained from the Shenyang Academy of Agricultural Sciences and the Dongya Seed Company, Shenyang, Liaoning Province, China, respectively. The seeds were sterilized with 1% sodium hypochlorite solution (v/v) for 10 min, rinsed with sterile water at least three times, and then soaked in distilled water for 12 h. One portion of the soaked seed was transferred to petri dishes lined with three filter papers, covered with gauze moistened with distilled water or aqueous CdCl_2_ solution (20 mg L^−1^), and placed in an incubator at 28 °C in the dark for 9 days to germinate. The germination percentage was calculated.

The other portion of soaked seed was transferred to petri dishes lined with three filter papers, covered with gauze moistened with distilled water or aqueous CdCl_2_ solution (20 mg L^−1^), and germinated for 3 days. The germinated seeds were transferred to plastic containers and cultured in quarter-strength Hoagland’s solution (pH 6.0) supplemented with 20 mg L^−1^ CdCl_2_ at 28 °C under a 16-h/8 h (light/dark) photoperiod for 3 or 6 days, and the light density was 200 μmol m^−2^ s^−1^. The control lacked cadmium. The coleoptile and radicle from germinated seeds at 3 days, and the shoot and root from seedlings at 6 and 9 days post-germination, were collected and stored directly in liquid nitrogen (for subsequent analyses of ROS, plant hormone, and sugar contents, and enzyme activities), or dried at 80 °C to constant weight (for biomass and Cd content determinations) before storage. Each treatment was replicated at least four times with three seedlings per replicate.

### Calculation of the relative growth rate

A seed was considered to have germinated when the radicle or coleoptile was at least 2 mm long. The germination percentage was calculated as the proportion of seed that had germinated after 3, 6, and 9 days in either water or Cd solution (20 mg L^−1^). The length of the plumules and radicles or shoots and roots of between 10 and 15 of the Cd-treated plantlets for each time period was measured with a ruler. The biomass was determined by measuring the dry weight of the plumules and radicles, or shoots and roots.

### Cd estimation

The plant tissue samples were washed with deionized water three times, and then were dried at 105 °C for 15 min and at 80 °C until constant weight. The dry plant tissues were ground to powder, and then 0.2 g of the dry powder was digested with HNO_3_ and HClO_4_ (83:17, v/v) for 24 h. The digested solutions were filtered after dilution with deionized water. Then the Cd concentration in the solutions was measured using an atomic absorption spectrophotometer (280Z, Agilent, USA).

### *Determination of O*_*2*_^*•−*^*, malondialdehyde, and proline concentrations and relative electrolyte leakage*

To determine the O_2_^•−^ concentration, a portion of the sample (2.0 g) was mixed with 3 mL of 65 nmol L^*−*1^ phosphate buffer (pH 7.8) and centrifuged for 10 min at 12,000 g. The supernatant (2.0 mL) was mixed with phosphate buffer (1.5 mL) and hydroxylamine hydrochloride (0.5 mL) at 25 °C for 20 min. Then, 2.0 mL of the reaction mixture was mixed with 17 mmol L^−1^ sulfanilic acid (2.0 mL) and 27 mmol L^−1^ α-naphthylamine (2.0 mL) at 30 °C for 30 min. The absorbance was measured at 530 nm using a UV-T6 spectrophotometer (V-5600, Shanghai Metash Instruments, China).

To determine the malondialdehyde (MDA) concentration, a sample (0.5 g) was mixed with 4.0 mL of 10% trichloroacetic acid and centrifuged at 5000 g for 10 min at 4 °C. The supernatant was assayed for MDA following the method described by Aravind and Prasad (2003). To determine the free L-proline concentrations, a sample (0.2 g) was mixed with 5 mL of 3% sulfosalicylic acid and extracted in a boiling water bath for 10 min, and then centrifuged at 1000 g for 10 min. The supernatant was measured using the method described by Bates and Waldren (1973). The relative electrolyte leakage rate was measured following the procedure described by Bajji et al. (2002).

### Determination of plant hormone contents

Fresh tissue (1 g) was ground to powder in liquid nitrogen and extracted with 10 mL of 80% precooled methanol at 4 °C for 12 h. The residue was extracted with 5 mL of 80% precooled methanol for 15 min. The supernatant from the combined extracts was collected by centrifuging at 12,000 g for 10 min. The supernatant was extracted with ethyl acetate three times, then dissolved in methanol, and stored at − 20 °C. The contents of GA_3_ and ABA were measured using the method described by Jia et al. (2020).

### Determination of sugar concentrations

Dried samples were powdered and homogenized in 80% ethanol, boiled at 70 °C for 30 min, and centrifuged at 8000 g at 4 °C for 10 min. The supernatant was collected, the residue was repeatedly extracted with 80% ethanol, and all supernatants were combined. The supernatant was decolorized with activated carbon by incubation in a water bath at 80 °C for 30 min, then adjusted to a constant volume, and filtered. A sample (1 mL) of the filtrate was mixed with 5 mL anthrone reagent and incubated in a boiling water bath for 10 min. After cooling, the total soluble sugar concentration in the supernatant was measured following the method described by McCready et al. (1950). The fructose, glucose, and sucrose concentrations were measured using high-performance liquid chromatography (1525–2489, Waters, USA) using the method of Sanchez-Linares et al. (2012).

### Enzyme activity assay

The activity of lipoxygenase (LOX) was determined using the procedure described by Surrey (1964). Fresh tissue (1 g) was ground to powder and homogenized in 50 mM Hepes (pH 7.0), 5 mM cysteine, and 10 mM EDTA. The mixture was centrifuged at 10,000 g at 4 °C for 20 min. The supernatant was assayed for enzyme activity.

To determine the activities of antioxidant enzymes, samples of fresh plant tissue (500 mg) were homogenized in 5 mL of 100 mM pre-cooled phosphate buffer (pH 7.5) containing 1 mM EDTA. The homogenate was centrifuged at 12,000 g for 15 min at 4 °C. The activities of antioxidant enzymes in the supernatant were determined. The SOD activity was measured spectrophotometrically at 560 nm as described by Tewari et al. (2008). The POD activity was measured as guaiacol oxidation of H_2_0_2_ at 470 nm in accordance with the method described by Lacan and Baccou (1998). The CAT activity was determined by measuring the decrease in H_2_O_2_ concentration at 240 nm as described by Ishibashi et al. (2008).

To measure the activities of sucrose metabolism enzymes, fresh plant tissue (1 g) was ground to powder with quartz sand and homogenized in 50 mM phosphate buffer (pH 7.5) at 4 °C. After centrifugation at 12,000 g for 20 min at 4 °C, the supernatant was divided into two portions: one portion was analyzed for the activities of sucrose phosphate synthase (SPS), sucrose synthase (SS), and neutral invertase (NI) following the procedure described by Saher et al. (2005), and the second portion was stored at − 80 °C. The precipitate was resuspended in the same extraction buffer with 1 M KCl and agitated continuously for 60 min at 4 °C. The homogenate was centrifuged at 12,000 g for 20 min, and the supernatant was mixed with the stored supernatant solution. The acid invertase (AI) activities of the mixture was determined using the method of Saher et al. (2005).

### RNA extraction and analysis

Total RNA was isolated from tissues using the RNA Pure Plant Kit (Qiagen, Germany). Purified RNA (about 2 μg) was reverse transcribed to synthetic cDNA using MMLV reverse transcriptase (Promega, USA). Quantitative reverse-transcription PCR (qRT-PCR) assays were performed with an ABI 7500 real-time PCR detection system (Applied Biosystems, USA) using the SuperReal PreMix Plus Kit (Qiagen, Germany). The reaction protocol was 40 cycles of 95 °C for 10 s (denaturation), 60 °C for 20 s (annealing), and 72 °C for 30 s (extension). The ΔΔ^*C*t^ method was used to calculate the transcript levels of the relevant genes (Livak and Schmittgen 2001). The primers used to amplify the *SOD* gene were 5′-CGGTGCACCAGAAGACGAAG-3′ and 5′-GCCAGTCTTCCACCAGCATT-3′, and the product size was 198 base pair (bp). The *CAT* gene primers were 5′-TCCCAACTACCTGATGCTGC-3′ and 5′-GTTGGGCTTGCGTATGGTTG-3′, and the product size was 209 bp. The *POD* gene primers were 5′-TGGAACACAAGCACGAACCC-3′ and 5′-CCTTCCACAGCGTCTCGTT-3′, and the product size was 279 bp (Ramazan et al. 2021). The *ZmTubulin1* (ID: Zm00001d013367) gene was used as an internal control. The primers used to amplify *ZmTubulin1* were 5′-GTGTCCTGTCCACCCACTCTCT-3′ and 5′-GGAACTCGTTCACATCAACGTTC-3′, and the product size was 299 bp (Tian et al. 2019).

### Statistical analysis

All data were presented as means and the standard deviation (SD). One-way analysis of variance (ANOVA) and Duncan’s multiple-range test were conducted using SPSS version 26. The significance level applied was *P* < 0.05. Each experiment comprised four replicates.

## Result and discussion

### Effects of Cd accumulation on germination and seedling biomass

**Fig. 1:**
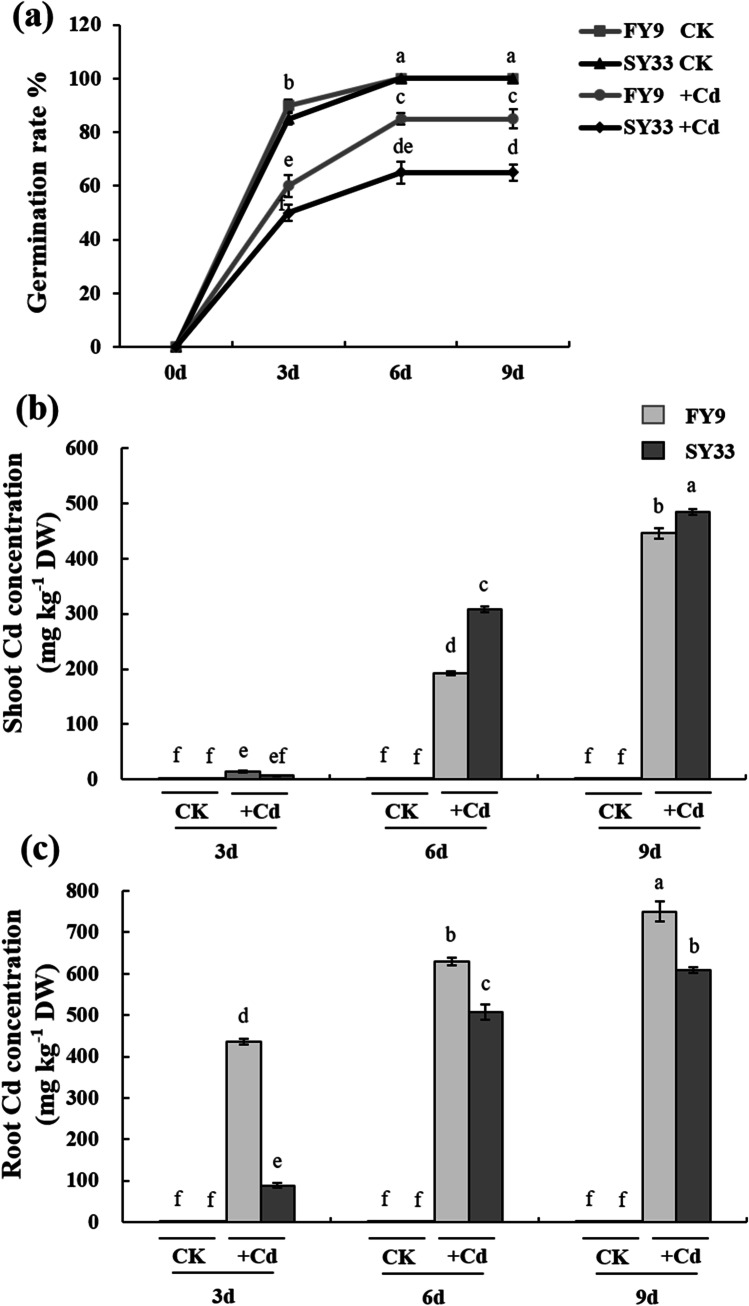
Seed germination percentage and cadmium (Cd) concentration in seedlings of two maize varieties under exposure to 20 mg L^−1^ CdCl_2_. **a** Germination percentage, **b** Cd concentration in the shoots, and **c** Cd concentration in the roots. The data are the means ± SD (*n* = 4). Different lowercase letters indicate significant differences (Duncan’s multiple-range test, *P* < 0.05). The experiments were repeated four times

We examined the influence of Cd on the germination of two maize varieties, FY9 and SY33, which were selected on account of their response to Cd stress from among 16 varieties that are widely grown in northeast China. The germination percentage of FY9 and SY33 seed treated with 20 mg L^−1^ Cd was 15% and 36% lower than the germination percentage of the control seed, respectively (Fig. 1a). The growth of the plumules and radicles of the seed treated with Cd for 3 days was inhibited. Growth of the shoots and roots of seedlings treated with Cd for 6 and 9 days was less than that of the control seedlings (Fig. S1). The shoots and roots of SY33 were noticeably shorter than those of FY9 (Table S1). The biomass accumulation was also reduced by Cd treatment and followed a similar pattern to that of the shoot and root lengths (Table 1). Many researchers have reported that heavy metal toxicity inhibits seed germination (Kuriakose and Prasad 2008; Junyu et al. 2008; Sfaxibousbih et al. 2010). The Cd that accumulates in the germinating seed and seedling impairs many metabolic processes in cells, with effects on the germination percentage and biomass accumulation (Zhang et al. 2013). We observed that the germination percentage, biomass, and growth, including the lengths of the shoots and roots, of the two maize varieties decreased in response to Cd treatment, which is consistent with previous results (Sfaxibousbih et al. 2010; Xu et al. 2014; Guo et al. 2019). Cadmium had a greater effect on SY33 than on FY9, and SY33 was more sensitive to Cd than FY9, which is consistent with our previous research (Li et al. 2020).

**Table 1:** The biomass (dry weight) of the two maize varieties

Treatment time (day)	Treatment	Variety	Biomass (DW)	
			Shoot (mg)	Root (mg)
3	CK	FY9	2.4 ± 0. 3e	2.2 ± 0. 11f
		SY33	2.3 ± 0.19e	2.1 ± 0.31f
	Cd	FY9	1.5 ± 0.14e	1.5 ± 0.2f
		SY33	1.1 ± 0.06e	1.1 ± 0.03f
6	CK	FY9	53.2 ± 1.3b	21.2 ± 1.3b
		SY33	51.2 ± 0.89b	19.3 ± 0.84b
	Cd	FY9	28.4 ± 0.92c	12.3 ± 0.91d
		SY33	20.4 ± 0.6d	8.2 ± 0.59e
9	CK	FY9	104.4 ± 0.66a	31.6 ± 1.2a
		SY33	101.5 ± 2.1a	30.2 ± 1.1a
	Cd	FY9	51.3 ± 4.7b	20.3 ± 0.66b
		SY33	22.4 ± 1.1d	14.1 ± 0.57c

The data shown are the means ± SD (*n* = 4). The different lowercase letters indicate significant differences by Duncan analyzed at *P* < 0.05. The experiments were repeated four times

The relationship between Cd accumulation and growth inhibition was investigated by determining the Cd concentrations. The Cd concentrations in two maize lines increased as the duration of Cd treatment increased, and the Cd concentrations in the shoot of FY9 were higher than those in SY33 and that were opposite in the root. Higher concentrations of Cd ions were detected in the root of the Cd-treated material, indicating that, as the primary line of defense, the root deposits, chelates, and stores the majority of Cd that is absorbed from the soil, and the remainder is transported to the shoot by metal ion transporters. The accumulation of Cd in the root of FY9 was higher than that in SY33, but less in the shoot. These results indicated that FY9 has superior abilities to deposit and chelate Cd than SY33.

### Effect of Cd on redox homeostasis in the seed during germination

**Fig. 2:**
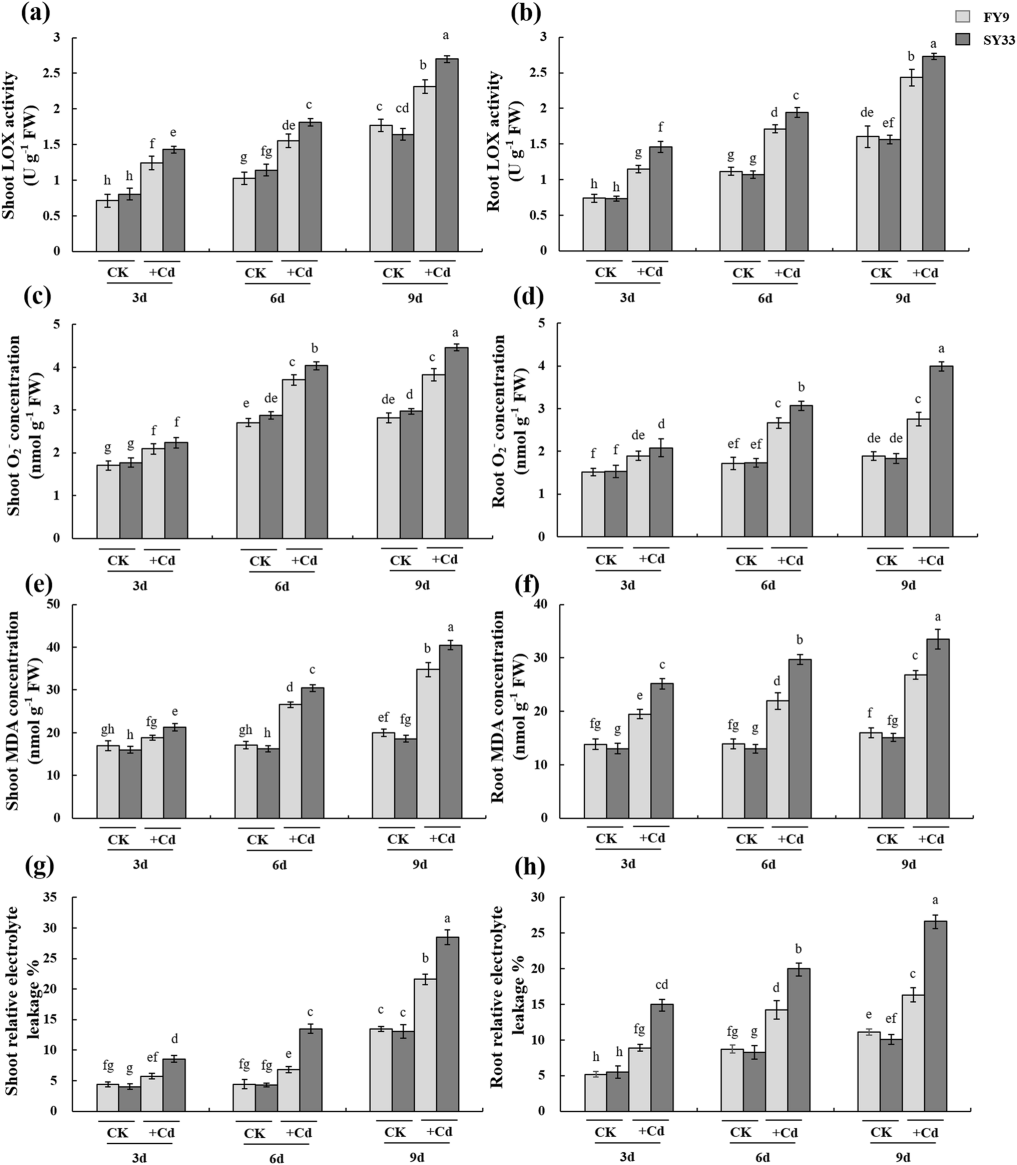
Indices of oxidative damage in shoots and roots of two maize varieties under exposure to 20 mg L^−1^ CdCl_2_. **a** Lipoxygenase (LOX) activity in shoots, **b** LOX activity in roots, **c** superoxide (O_2_^•−^) concentration in shoots, **d** O_2_^•−^ concentration in roots, **e** malondialdehyde (MDA) concentration in shoots, **f** MDA concentration in roots, **g** relative electrolyte leakage in shoots, and **h** relative electrolyte leakage in roots. The data are the means ± SD (*n* = 4). Different lowercase letters indicate significant differences (Duncan’s multiple-range test, *P* < 0.05). The experiments were repeated four times

The Cd stress induced oxidative stress in the seedlings. The concentrations of the main indices of oxidative damage, i.e., LOX, O_2_^•−^, and MDA, increased noticeably with prolonged duration of Cd treatment (Fig. 2a–f). With 20 mg L^−1^ Cd treatment for 9 days, the LOX activities of FY9 and SY33 were 23.69% and 35.45% higher than those in CK in the leaves, and 34.06% and 42.85% higher in the roots; the O_2_^•−^ concentrations of FY9 and SY33 were 26.40% and 36.86% higher than those in CK in the leaves, and 31.53% and 54.10% higher in the roots; and the MDA concentrations of FY9 and SY33 were 42.51% and 53.08% higher than those in CK in the leaves, and 41.13% and 54.98% higher in the roots, respectively. Higher quantities of ROS accumulated in Cd-treated SY33 than in Cd-treated FY9. The relative conductivities also increased in Cd-treated plants, with a greater increase observed in SY33 than in FY9 (Fig. 2g and h). Accumulation of Cd ions in cells causes alteration of the cellular metabolic balance, and the generation of ROS is stimulated, causing oxidative stress and inhibiting plant growth and development (Gill and Tuteja 2010). A major species of ROS, O_2_^•−^, is generated by xanthine oxidase and NADPH-dependent oxidase that are induced by heavy metals such as Cd (Rio et al. 2006; Rodriguezserrano et al. 2006). Lipoxygenase induces lipid peroxidation, which in turn generates MDA in plants (Liang et al. 2003; Montillet et al. 2004). The LOX activity, and O_2_^•−^ and MDA concentrations are good indicators of the damage caused by Cd-induced oxidative stress. These results indicated that Cd caused more damage to SY33 than to FY9. Plants produce antioxidants as a means of coping with Cd-induced oxidative stress (Liang et al. 2003; Guo et al. 2017).

**Fig. 3:**
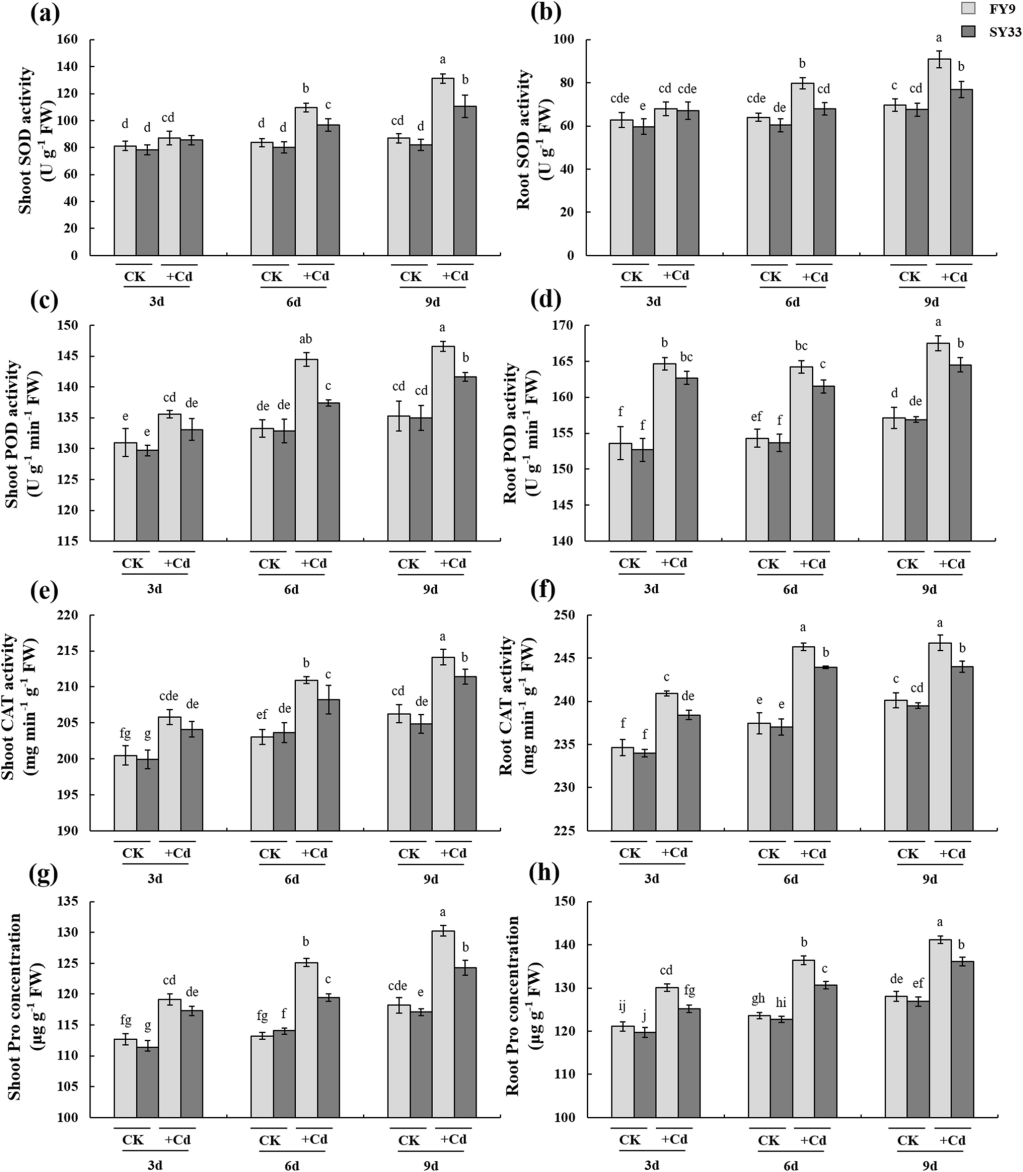
**a** Superoxide dismutase (SOD) activity in shoots, **b** SOD activity in roots, **c** peroxidase (POD) activity in shoots, **d** POD activity in roots, **e** catalase (CAT) activity in shoots, **f** CAT activity in roots, **g** proline (Pro) concentrations in shoots, and **h** proline concentrations in roots. The data are the means ± SD (*n* = 4). Different lowercase letters indicate significant differences (Duncan’s multiple-range test, *P* < 0.05). The experiments were repeated four times

Plants produce antioxidants as a means of coping with Cd-induced oxidative stress (Liang et al. 2003; Guo et al. 2017). We also examined how the antioxidative ability differed in Cd-treated FY9 and SY33. The activities of antioxidative enzymes significantly increased in the leaves and roots with prolonged duration of Cd treatment (Fig. 3a–f). Under 20 mg L^−1^ Cd treatment, the SOD activities in the shoots of FY9 and SY33 were 33.71% and 22.07% higher than those in CK, whereas those in the roots of FY9 and SY33 were 15.97% and 9.46% higher than those in CK, respectively. The activities of POD were 4.98% and 2.83% higher in the shoots, and 3.19% and 1.84% higher in the roots, of FY9 and SY33 than those in CK, respectively. The activities of CAT were 3.66% and 2.63% higher in the shoots, and 2.68% and 1.84% higher in the roots, of FY9 and SY33 than those in CK, respectively. The activities of the antioxidative enzymes were generally higher in FY9 than in SY33. The enzymes SOD, POD, and CAT are representative enzymatic antioxidants that scavenge ROS induced by biotic and abiotic stressors. Proline accumulated in the shoots and roots of Cd-treated plants, with greater accumulation observed in FY9 than in SY33 (Fig. 3g and h). Proline, a representative non-enzymatic antioxidant and osmotic protectant, scavenges OH^−^ and ^1^O_2_, and protects the cellular backbone (Kaul et al. 2008).

Although more Cd accumulated in FY9 than in SY33, antioxidants were more active in FY9 than in SY33, indicating that less ROS accumulated, and the germination percentage and growth were superior in FY9 than in SY33. These factors may help to explain why FY9 was tolerant to, and SY33 was sensitive to, Cd stress.

### Effects of Cd on endogenous hormones in the seed during germination

The phytohormones ABA and GA regulate seed germination and responses to abiotic stress. We measured the concentrations of ABA and GA_3_ in Cd-treated plants to determine how these phytohormones were affected following seed germination. The ABA concentrations were 29.6% and 36.7% higher in the shoots of FY9 and SY33 treated with 20 mg L^−1^ CdCl_2_ for 6 days than those in CK, respectively, but did not change noticeably in the roots (Fig. S2a and b). The ABA concentrations in the shoot were higher in SY33 than in FY9. The GA_3_ concentrations in the shoots and roots of the two Cd-treated maize varieties were noticeably lower than in the CK (Fig. S2c and d). The data suggested that the accumulation of ABA was greater than that of GA_3_ in the Cd-treated plants, and that these hormones accumulated to higher concentrations in SY33 than in FY9.

Plant hormones regulate plant growth and development, and help plants respond to abiotic stresses. The hormones ABA and GA cooperate to regulate seed germination (Duval et al. 2012). The ABA and GA concentrations changed in response to Cd treatment (Guo et al. 2019). ABA regulates the transcription of resistance genes that respond to Cd stress. The concentrations of ABA in the leaves of FY9 and SY33 distinctly increased under Cd treatment; in previous studies, Chaca et al. (2014) and Guo et al. (2019) also observed increases in ABA under exposure to Cd in soybean and wheat.

### Effects of Cd on sucrose metabolism in the seed during germination

**Fig. 4:**
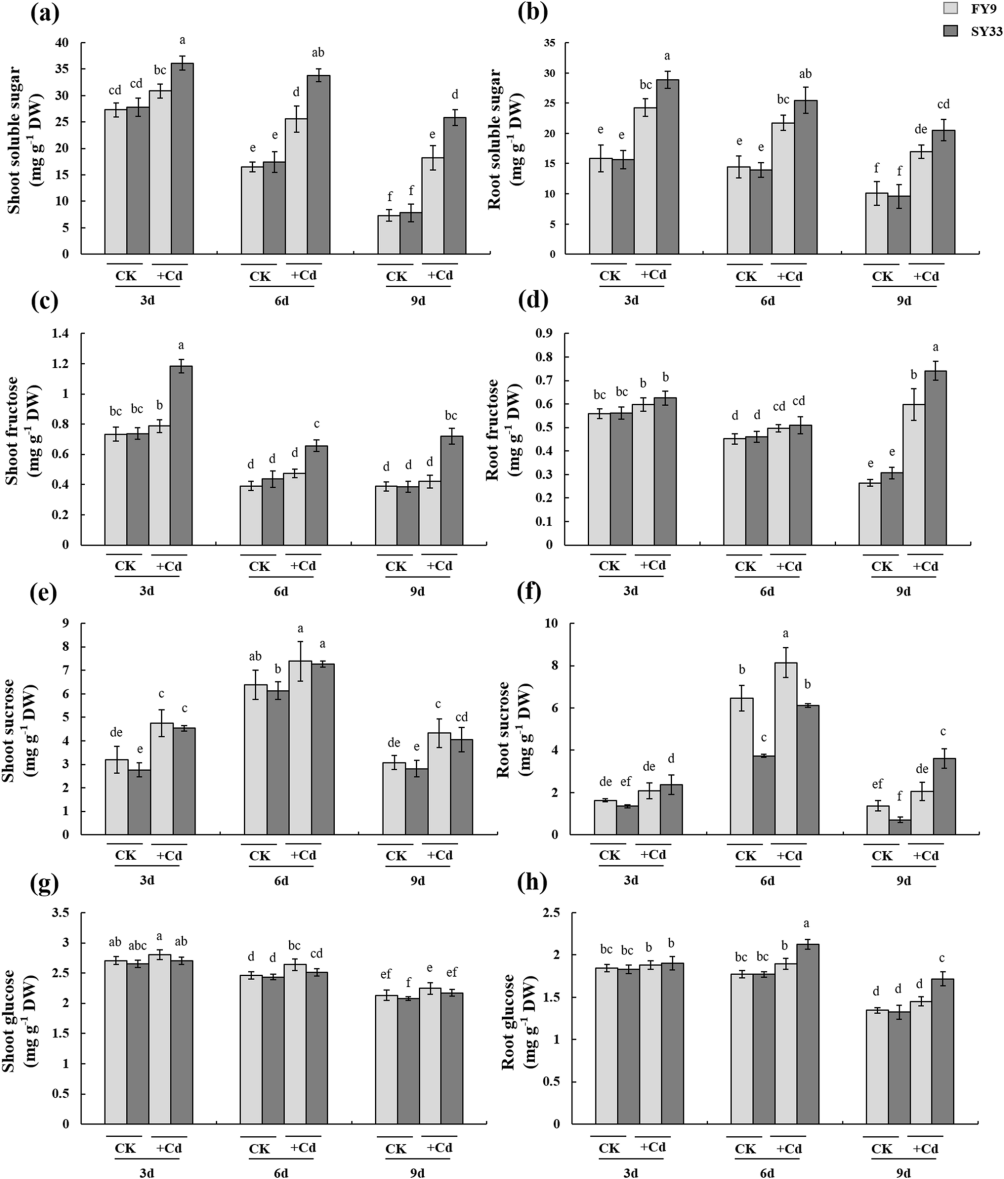
**a** Total soluble sugar concentrations in the shoots, **b** total soluble sugar concentrations in roots, **c** fructose concentrations in the shoots, **d** fructose concentrations in roots, **e** sucrose concentrations in the shoots, **f** sucrose concentrations in roots, **g** glucose concentrations in the shoots, and **h** glucose concentrations in roots. The data are the means ± SD (*n* = 4). Different lowercase letters indicate significant differences (Duncan’s multiple-range test, *P* < 0.05). The experiments were repeated four times

Sucrose metabolism is inhibited in Cd-stressed plants. In this study, we measured the sucrose metabolite concentrations as indices of sucrose metabolism. The total soluble sugar concentrations were increased considerably in the shoots and roots of FY9 and SY33 under Cd treatment, with greater accumulation of total soluble sugars in SY33 than in FY9, especially in the shoots (Fig. 4a and b). The fructose concentrations followed a similar pattern as the total soluble sugars, with greater fructose accumulation in SY33 than in FY9 (Fig. 4c and d). The concentrations of sucrose generally increased as the duration of Cd treatment increased and peaked in plants treated with 20 mg L^−1^ of Cd for 6 days (Fig. 4e and f). The concentrations of glucose did not change notably in the Cd-treated plants (Fig. 4g and h). The concentrations of sucrose, glucose, and fructose increased, and the concentrations of total soluble sugars increased notably under Cd treatment. These results indicated that higher amounts of sugars were used to maintain the osmotic balance under Cd stress, whereas fewer sugars were provided for plant growth under Cd treatment, especially in the Cd-sensitive SY33. Oxidative stress induced by Cd damages the cellular membrane and disrupts the osmotic balance in cells (Sharma and Dietz 2009; Gill and Tuteja 2010). The present results showed that oxidative stress was more severe in SY33 than in FY9; thus, higher quantities of soluble sugars accumulated in SY33 to counteract the Cd stress.

The activities of many enzymes that help to regulate sucrose metabolism are affected by Cd stress. The activities of SPS decreased in the shoots and roots as the duration of the Cd treatment increased (Fig. S3a and b). The activities of SS in the sucrose synthesis direction decreased and showed a greater decrease in SY33 than in FY9 (Fig. S3c and d). However, the activities of the sucrose hydrolysis enzymes AI, NI, and SS (in the hydrolysis direction) increased significantly in the shoots of Cd-treated seedlings, particularly in SY33 (Fig. S4a–f). These results suggested that, when treated with Cd, the production and accumulation of soluble sugars in germinating maize seed increased, especially in the Cd-sensitive variety SY33. The activities of sucrose metabolism enzymes were modulated by Cd. The activities of the sucrose enzymes in the synthesis direction decreased, whereas the activities of sucrose hydrolysis enzymes increased. Taken together, these results indicated that the antioxidant system actively protected the cells from damage by ROS, and sucrose metabolism passively maintained the basic environment for physiological activities in Cd-stressed plants. These two systems may be cooperative and complementary to help plants cope with Cd stress.

### Transcript levels of antioxidant enzymes in response to Cd stress during germination

**Fig. 5:**
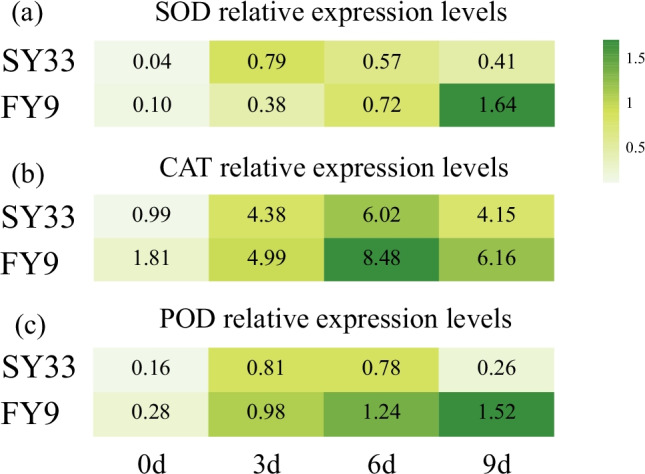
**a** Expression profiles of *SOD* genes, **b** expression profiles of *CAT* genes, and **c** expression profiles of *POD* genes. Data are the treatment means ± SD (*n* = 3). Different letters indicate significant differences (Duncan’s multiple-range test, *P* < 0.05)

Transcript levels of antioxidant enzymes are important indicators of the plant response to Cd stress. We measured the expression of *SOD* (Zm00001d031908), *CAT* (Zm00001d054044), and *POD* (Zm00001d040702) under 20 mg L^−1^ CdCl_2_ stress for 0, 3, 6, and 9 days. The transcript level of *SOD* increased gradually with the duration of Cd treatment in FY9, but decreased gradually at 6 days of Cd treatment in SY33 (Fig. 5a). The transcript level of *CAT* was significantly increased at 3 and 6 days of Cd treatment in FY9 and SY33, and decreased slightly at 9 days (Fig. 5b). The transcript level of *POD* significantly increased with duration of Cd treatment in FY9, but the gene was downregulated at 3 and 6 days in SY33 (Fig. 5c). These results suggested that the transcript levels of genes encoding antioxidant enzymes differed in response to Cd stress in the two maize varieties.

The changes in transcript level of antioxidant enzymes were similar to the changes in their activities. These findings may indicate that higher quantities of antioxidant enzymes were transcribed, translated, and accumulated in FY9 than in SY33 under Cd treatment. Therefore, the antioxidant abilities were higher and the levels of ROS were lower in FY9 than in SY33. ABA are involved in stress resistance to toxic metals (Hashem et al. 2014). As a signaling molecule, ABA might regulate the expression of stress resistance genes, including antioxidant enzyme genes, in response to Cd stress. Although the content of ABA increased in both maize varieties under Cd treatment, the trends in expression differed among antioxidant enzyme genes. This finding may be due to differences in responses to ABA signaling between the varieties.

In conclusion, with the accumulation of Cd in the germinating maize seed, the transcript levels and activities of antioxidases and proline concentrations increased to scavenge ROS, which help to alleviate oxidative damage in FY9 and SY33. The activities of antioxidants were higher, and the concentrations of ROS were lower, in FY9 and thus the cell damage caused by ROS was less severe. However, these responses were reversed in SY33. Therefore, sucrose metabolism that was induced to maintain the osmotic balance in damaged cells and to protect the plant from Cd stress was more active in SY33. The changes in sucrose metabolism allow greater quantities of sugars to be utilized to resist Cd stress and have fewer uses for growth, resulting in reduced biomass accumulation in SY33. These findings suggest that multiple systems are involved in the response to Cd stress, among which the antioxidant system and sucrose metabolism provide active and passive responses, respectively, in maize during seed germination.

## Supplementary Information

Below is the link to the electronic supplementary material.

Supplementary file1 (TIF 39888 KB)

Supplementary file2 (TIF 7560 KB)

Supplementary file3 (TIF 8108 KB)

Supplementary file4 (TIF 11497 KB)

Supplementary file5 (DOCX 2856 KB)

## Data Availability

The data and materials obtained in this study are available from the corresponding author on reasonable request.
