# Supplementary figures and images for: Changes in antioxidant system and sucrose metabolism in maize varieties exposed to Cd
Source: Environ Sci Pollut Res Int. 2022 Apr 28;29(43):64999–5011. doi: 10.1007/s11356-022-20422-8 (PMC9481512; doi:10.1007/s11356-022-20422-8)

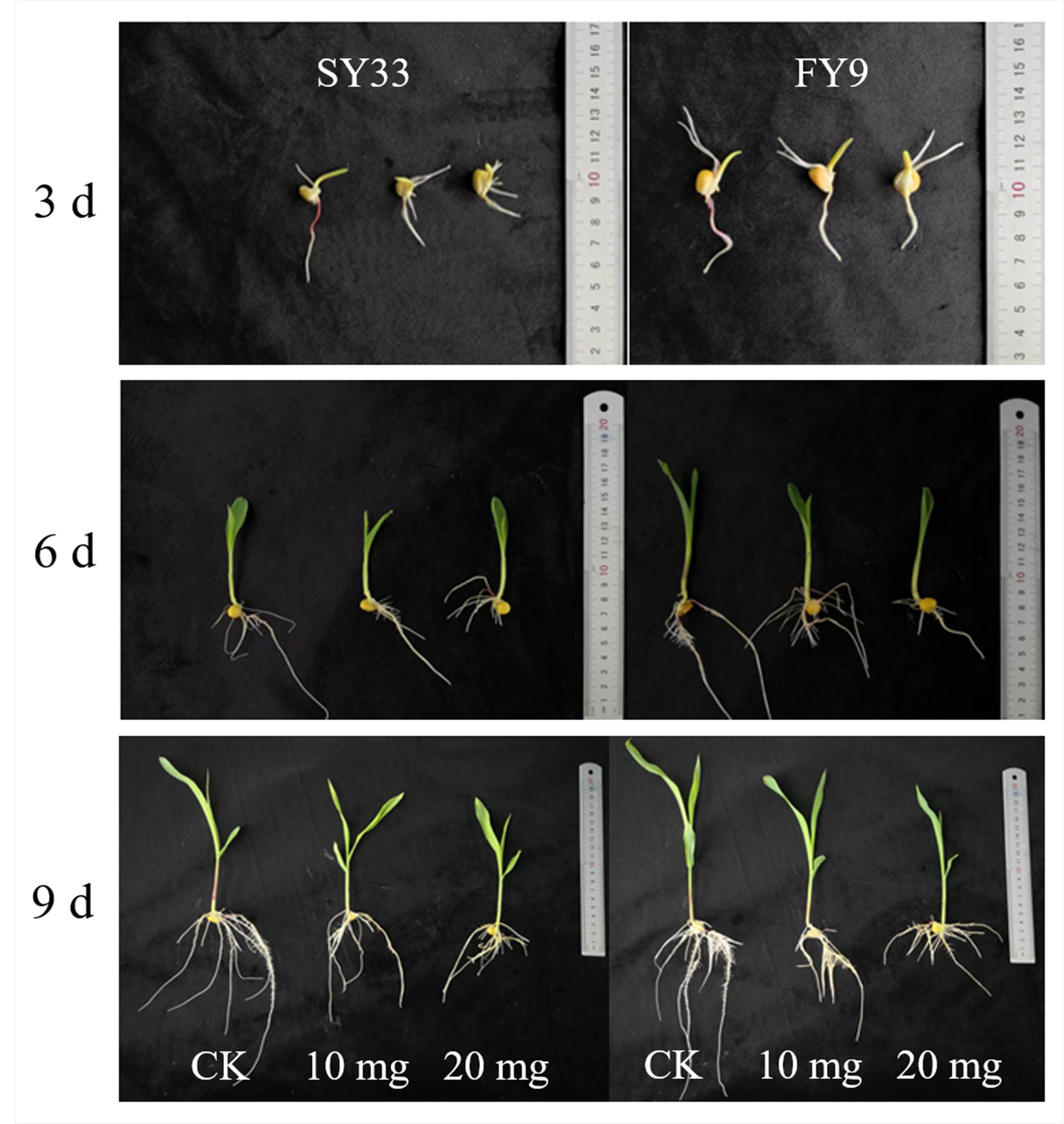

Supplement: Supplementary file 1 — Supplementary file1 (TIF 39888 KB) [file 11356_2022_20422_MOESM1_ESM.tif]

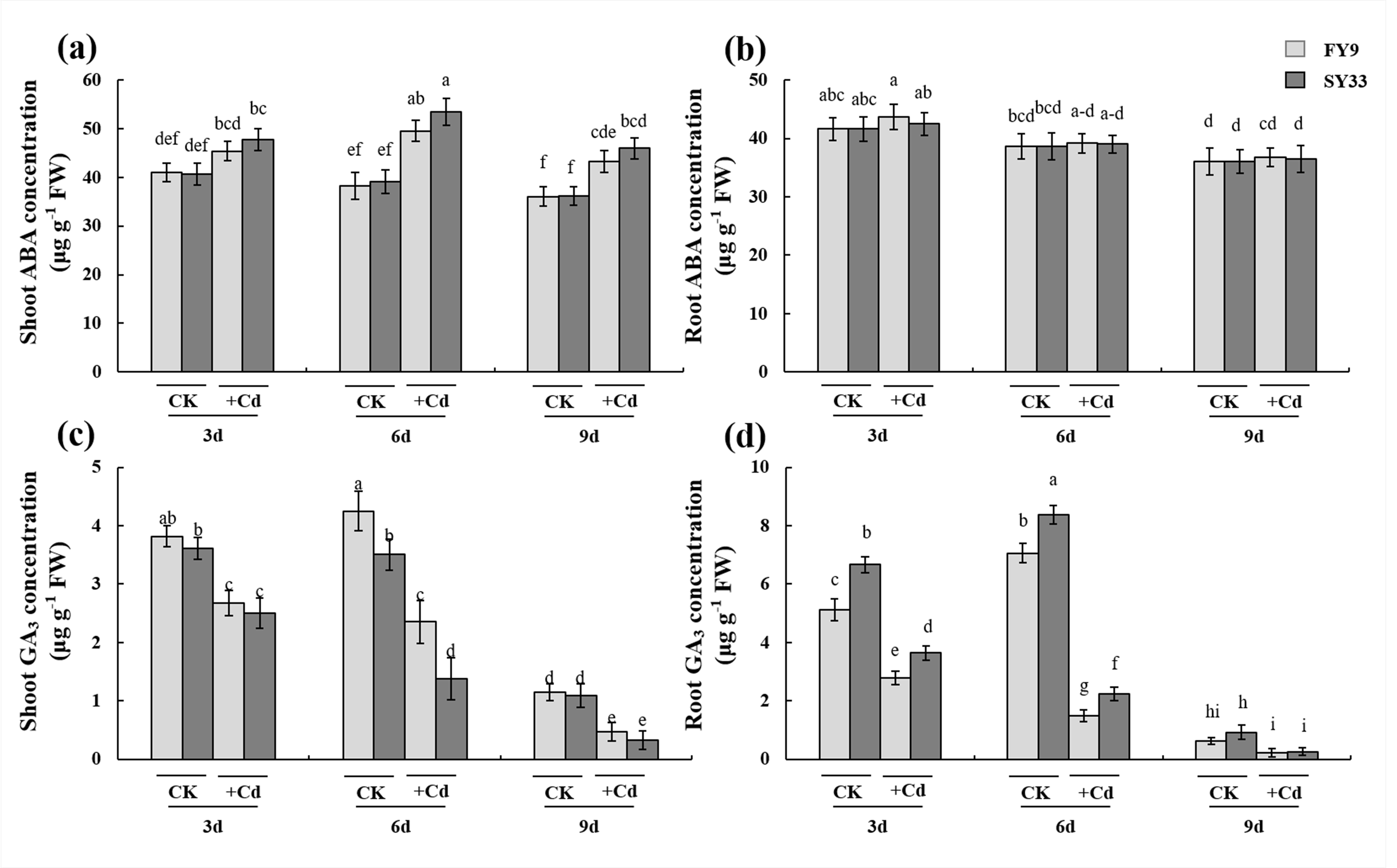

Supplement: Supplementary file 2 — Supplementary file2 (TIF 7560 KB) [file 11356_2022_20422_MOESM2_ESM.tif]

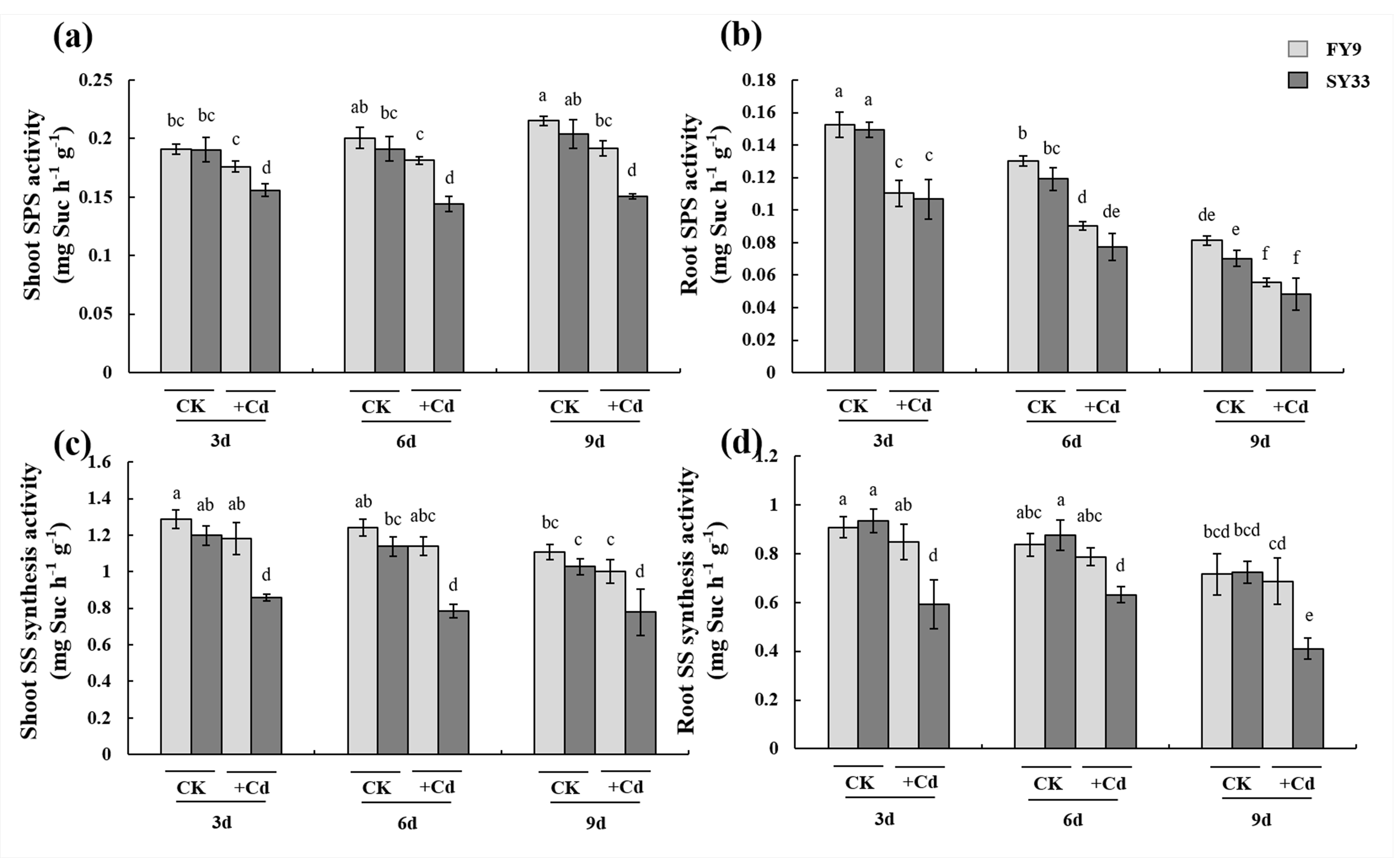

Supplement: Supplementary file 3 — Supplementary file3 (TIF 8108 KB) [file 11356_2022_20422_MOESM3_ESM.tif]

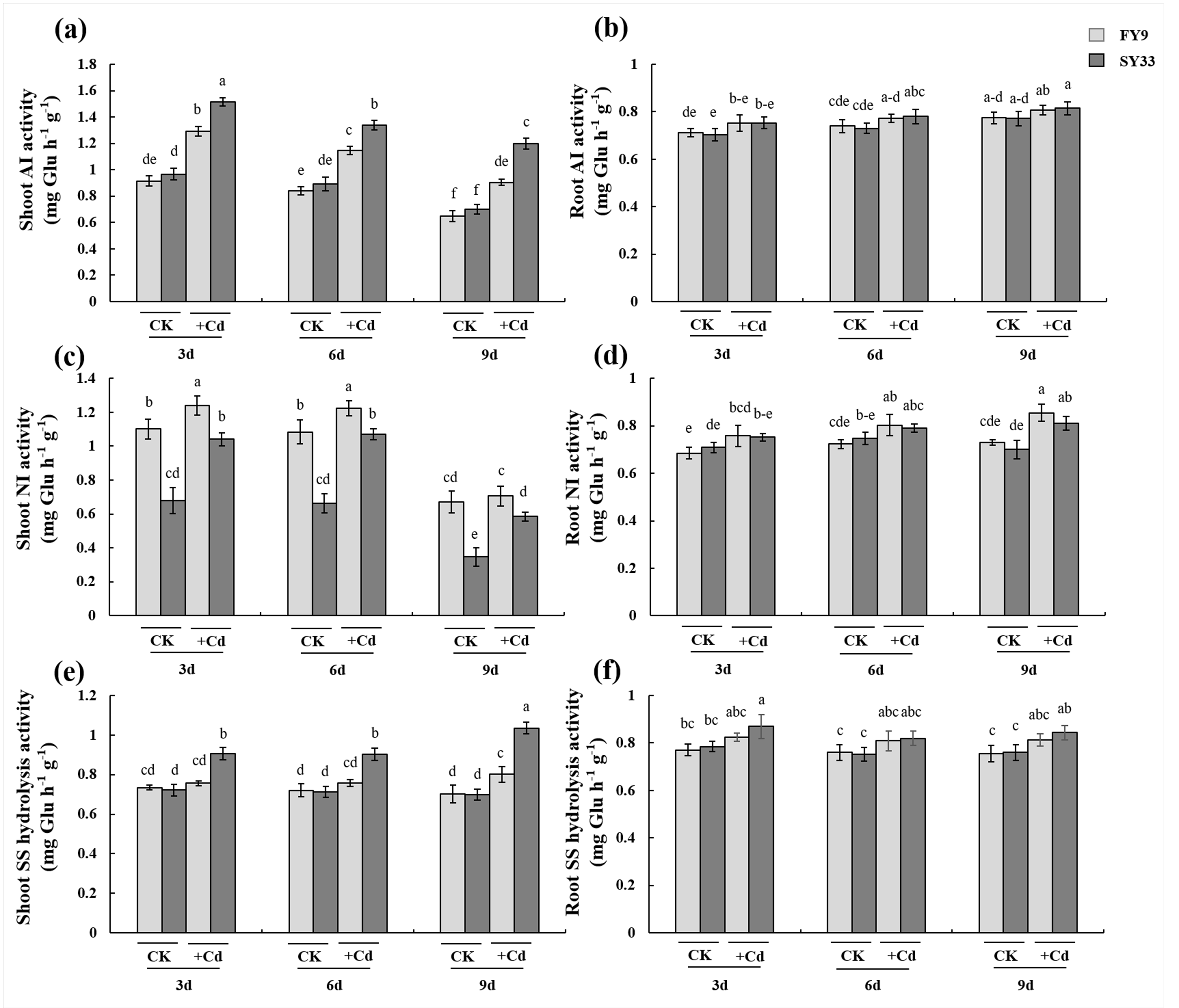

Supplement: Supplementary file 4 — Supplementary file4 (TIF 11497 KB) [file 11356_2022_20422_MOESM4_ESM.tif]
