# Supplementary material for: Changes in antioxidant system and sucrose metabolism in maize varieties exposed to Cd
Source: Environ Sci Pollut Res Int. 2022 Apr 28;29(43):64999–5011. doi: 10.1007/s11356-022-20422-8 (PMC9481512; doi:10.1007/s11356-022-20422-8)
Supplement: Supplementary file 5 — Supplementary file5 (DOCX 2856 KB) [file 11356_2022_20422_MOESM5_ESM.docx]

Changes in antioxidant system and sucrose metabolism in maize varieties exposed to Cd

Cong Li ^1^, Yingdi Cao ^1^, Meiyu Guo ^1^, Xinglin Ma ^2^, Yanshu Zhu ^1^, Jinjuan Fan ^1,^ ✉

^1^ College of Biological Science and Technology, Shenyang Agricultural University, Shenyang Key Laboratory of Maize Genomic Selection Breeding, Shenyang 110866, China

^2^ Institute of Crop Science, Chinese Academy of Agricultural Sciences (CAAS), Beijing, 100081, China

✉Corresponding author. E-mail: [jinjuanf@hotmail.com](mailto:jinjuanf@hotmail.com)

Table S1

Plant heights and root lengths of the two maize varieties

| **Treatment**  **time (d)** | **Treatment** | **Variety** | **Plant height (cm)** | **Root length (cm)** | |
| --- | --- | --- | --- | --- | --- |
| 3 | CK | FY9 | 1.87±0.067h | | 3.64±0.32fg |
|  |  | SY33 | 1.77±0.088h | | 3.57±0.18fg |
|  | Cd | FY9 | 1.30±0.058hi | | 2.60±0.10gh |
|  |  | SY33 | 0.83±0.088i | | 2.03±0.18h |
| 6 | CK | FY9 | 13.44±0.088cd | | 11.00±0.51b |
|  |  | SY33 | 12.93±0.536d | | 9.97±0.32b |
|  | Cd | FY9 | 8.00±0.29f | | 6.40±0.31d |
|  |  | SY33 | 6.00±0.12g | | 4.87±0.32e |
| 9 | CK | FY9 | 17.76 ± 0.33a | | 12.83 ± 0.10a |
|  |  | SY33 | 14.00 ± 0.44c | | 12.55 ± 0.60a |
|  | Cd | FY9 | 15.87 ± 0.49b | | 8.13 ± 0.35c |
|  |  | SY33 | 9.03 ± 0.41e | | 4.43 ± 0.31ef |

The data shown are the means ± SD (n=4). The different lowercase letters indicate significant differences by Duncan analyze at *P*<0.05. The experiments were repeated four times.


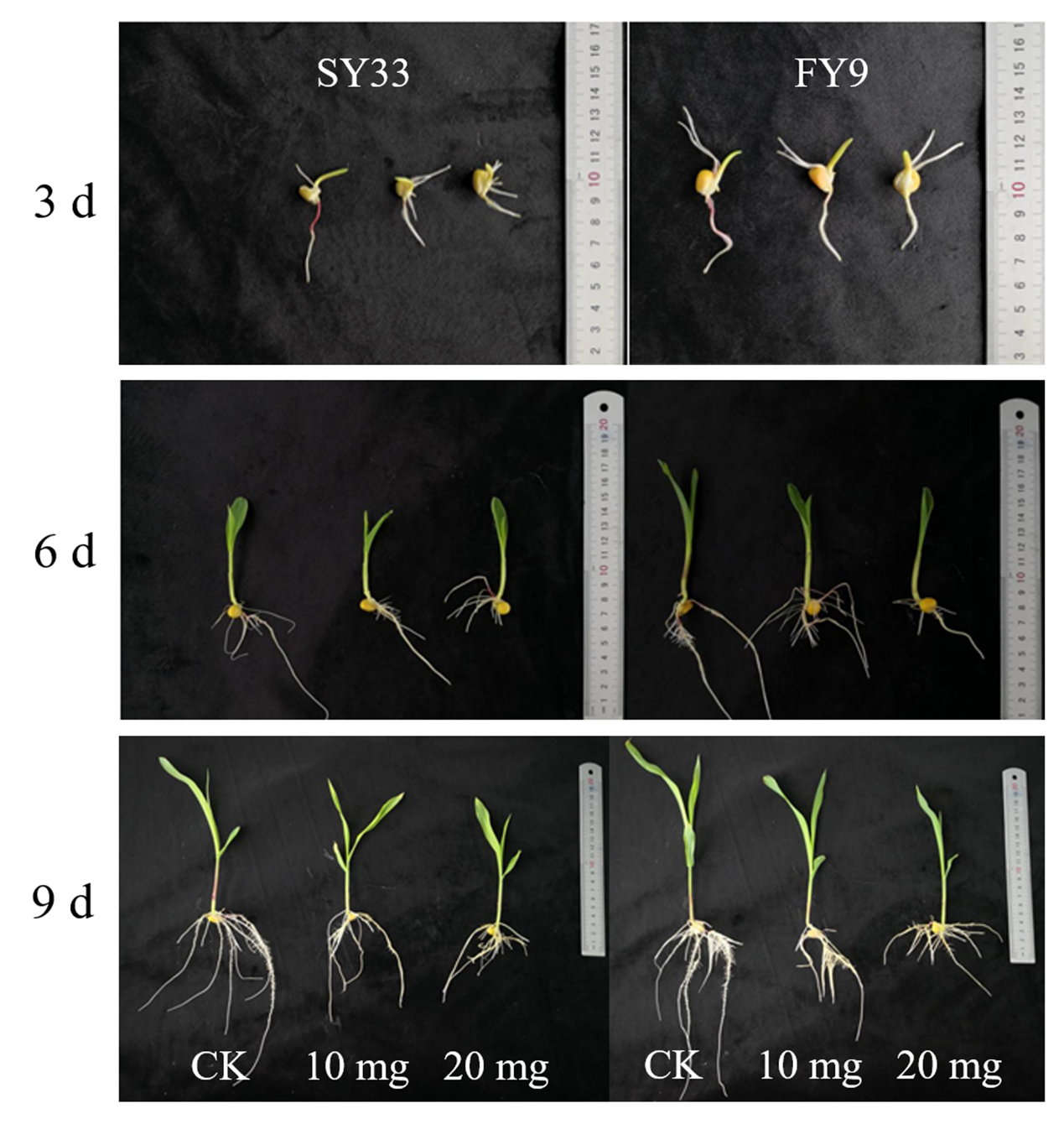


Fig. S1 Cd tolerance assay of SY33 and FY9. SY33 and FY9 were germinated under 0, 10 and 20 mg L^-1^ Cd treatments for 3, 6 and 9 d, respectively. CK indicate 0 mg L^-1^ Cd treatment. Scale bar: 20 cm.


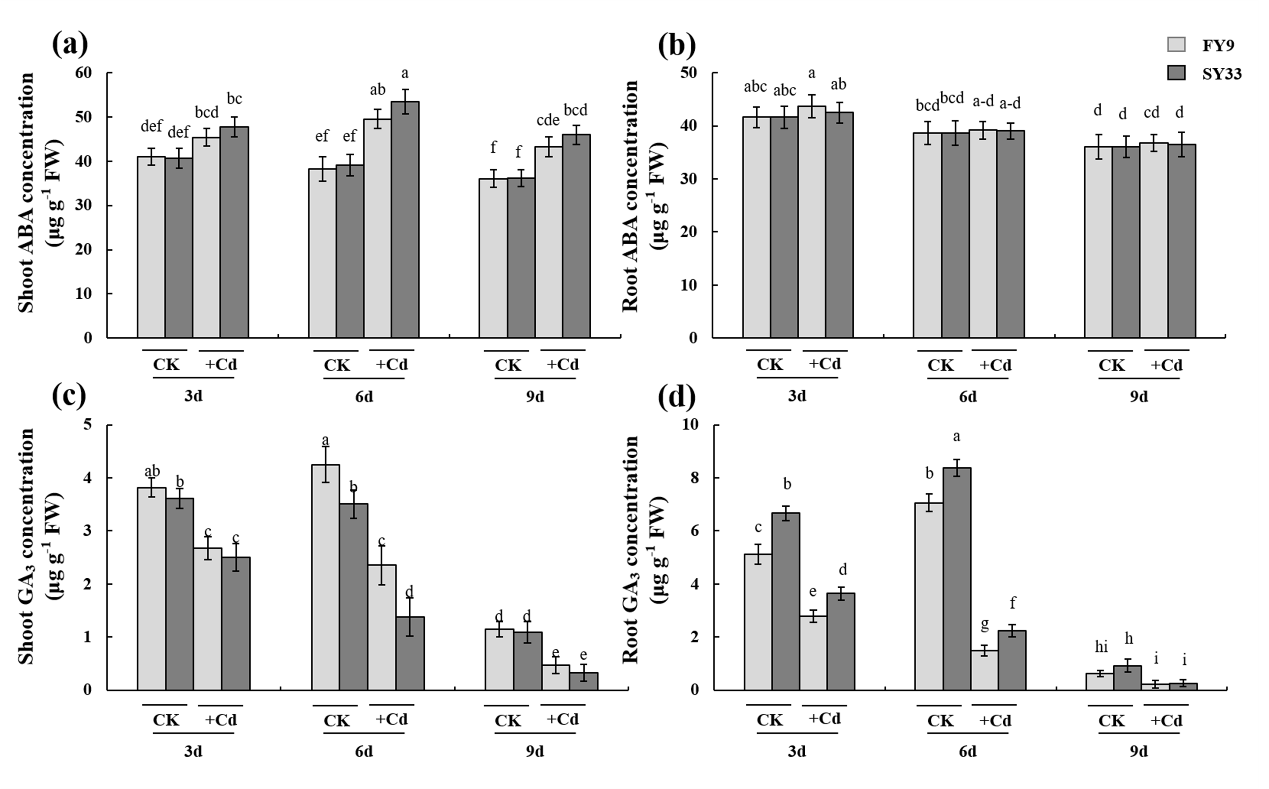


Fig. S2 a ABA concentration in shoots at 20 mg L^−1^ Cd concentrations, b ABA concentration in roots at 20 mg L^−1^ Cd concentrations, c GA3 concentrations in shoots at 20 mg L^−1^ Cd concentrations, d GA3 concentrations in roots at 20 mg L^−1^ Cd concentrations. The data shown are the means ± SD (n=4). The different lowercase letters indicate significant differences by Duncan analyze at P<0.05. The experiments were repeated four times.


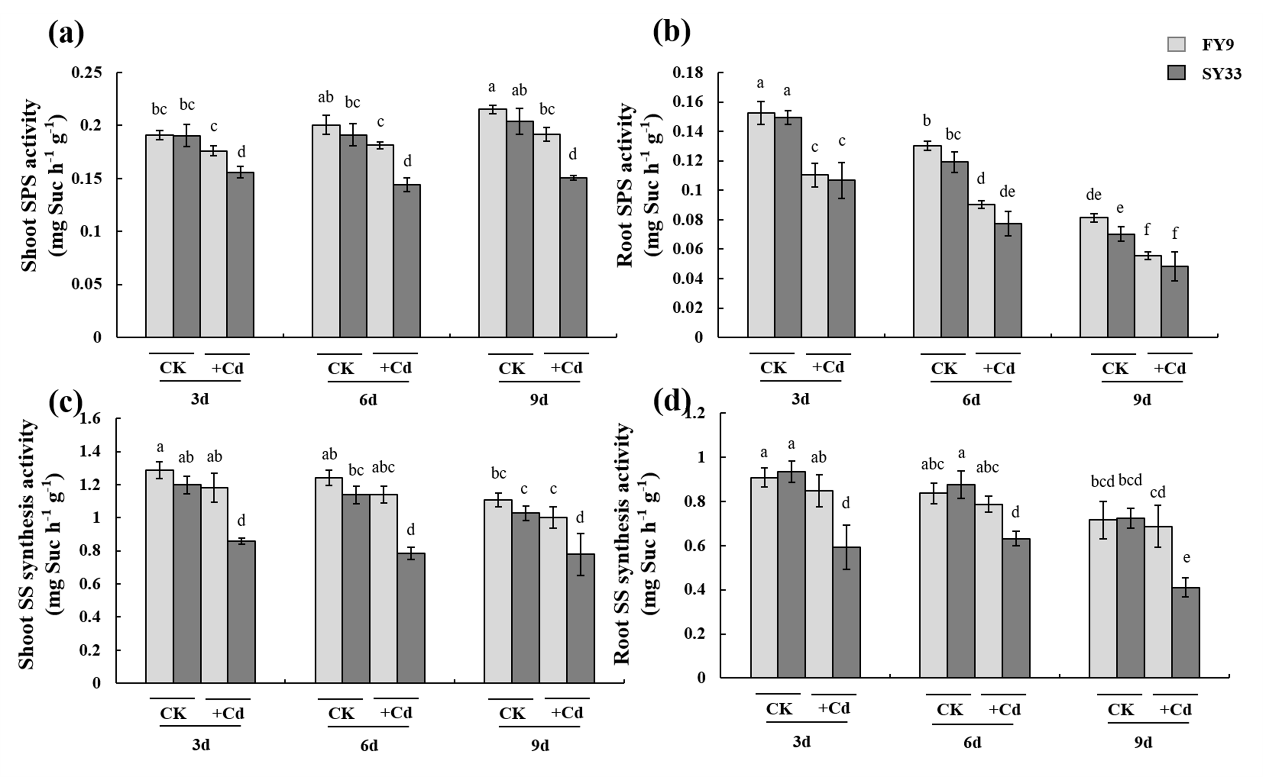


Fig. S3 **a** SPS activity in shoots under 20 mg L^−1^ Cd treatment, **b** SPS activity in roots under 20 mg L^−1^ Cd treatment, **c** SS synthesis activity in shoots under 20 mg L^−1^ Cd treatment, **d** SS synthesis activity in roots under 20 mg L^−1^ Cd treatment. The data shown are the means ± SD (n=4). The different lowercase letters indicate significant differences by Duncan analyze at *P*<0.05. The experiments were repeated four times.


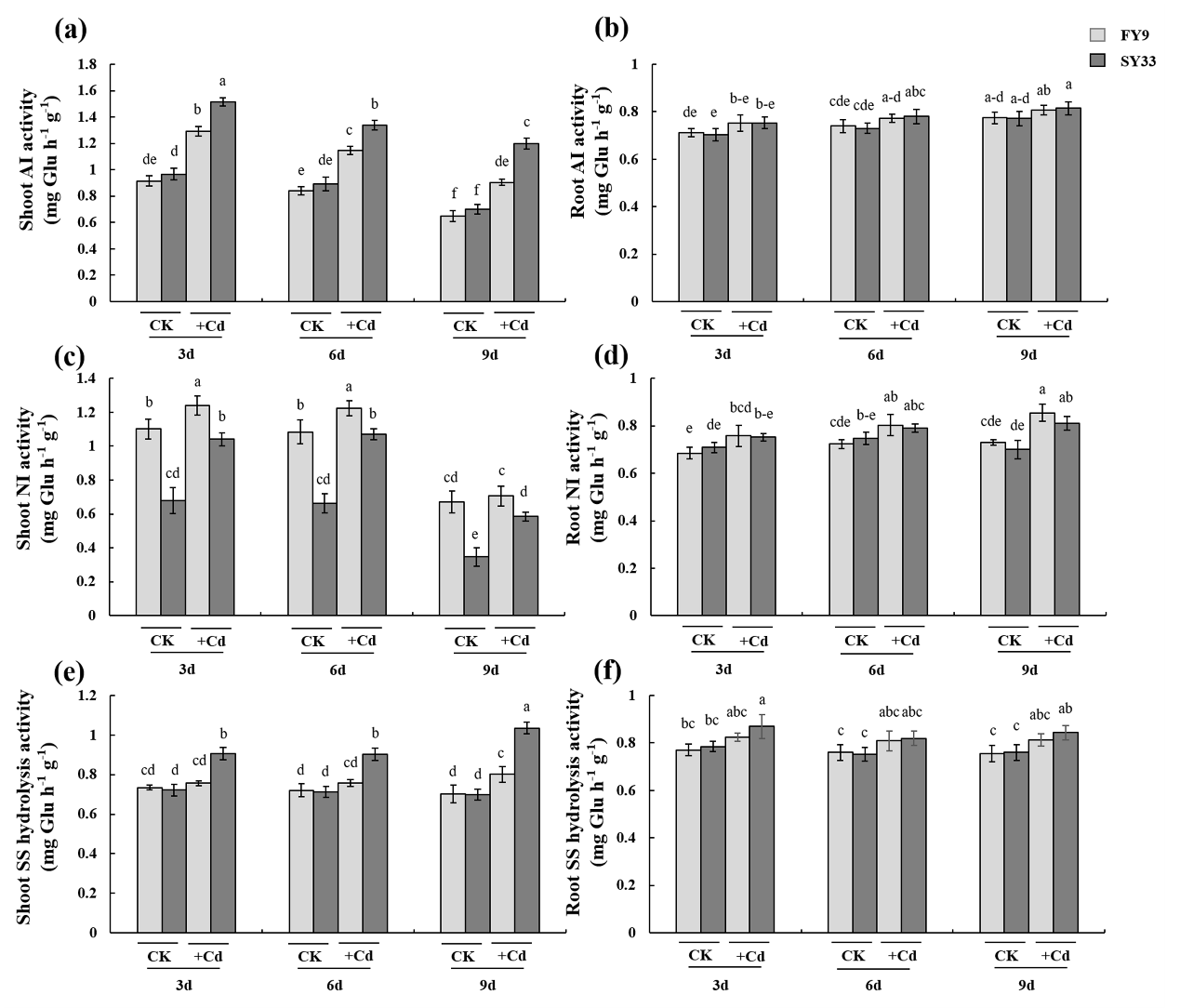


Fig. S4 **a** AI activity in shoots at 20 mg L^−1^ Cd treatment, **b** AI activity in roots at 20 mg L^−1^ Cd treatment, **c** NI activity in shoots at 20 mg L^−1^ Cd treatment, **d** NI activity in roots at 20 mg L^−1^ Cd treatment, **e** SS hydrolysis activity in shoots at 20 mg L^−1^ Cd treatment, **f** SS hydrolysis activity in roots at 20 mg L^−1^ Cd treatment. The data shown are the means ± SD (n=4). The different lowercase letters indicate significant differences by Duncan analyze at *P*<0.05. The experiments were repeated four times.
